# Brainstem tuberculoma mimicking brainstem stroke: Crossed syndrome in a young female

**DOI:** 10.5339/qmj.2026.18

**Published:** 2026-03-23

**Authors:** Mohammad Saquib Alam, Khwaja Saifullah Zafar, Ruhi Khan

**Affiliations:** 1 Department of Medicine, J.N. Medical College, Aligarh Muslim University, Aligarh, India *Email: msalam109@myamu.ac.in

**Keywords:** Brainstem tuberculoma, hemiparesis, cranial nerve palsy, MRI, MRS, India

## Abstract

**Introduction:**

Tuberculosis (TB) involving the central nervous system (CNS) can present as tuberculoma and may mimic neoplasms or vascular lesions, particularly when the brainstem is involved. Early recognition is critical in endemic settings such as India.

**Case presentation:**

A previously healthy female in late adolescence presented with a one-month history of headache followed by progressive left-sided weakness and multiple cranial nerve deficits, producing a crossed brainstem syndrome. Magnetic resonance imaging (MRI) of the brain revealed conglomerated ring-enhancing lesions in the midbrain and pons, accompanied by surrounding edema. Magnetic resonance spectroscopy (MRS) demonstrated a lipid–lactate peak. Cerebrospinal fluid (CSF) analysis and systemic laboratory investigations were within normal limits. Empirical anti-tubercular therapy (ATT) with adjunctive corticosteroids was initiated, with clinical improvement noted within two weeks and continued gains on follow-up.

**Discussion:**

Brainstem tuberculoma can closely mimic brainstem stroke and other mass lesions. In endemic regions, characteristic MRI/MRS findings should prompt consideration of tuberculoma even when CSF findings are normal. Early treatment may prevent the need for invasive diagnostic procedures and improve outcomes.

**Conclusion:**

Brainstem tuberculoma should be considered an important differential diagnosis in young patients presenting with crossed brainstem signs in TB-endemic regions. A combination of characteristic imaging findings and high clinical suspicion can support the early initiation of ATT with adjunctive corticosteroids, which, in this case, was associated with prompt and favorable neurological recovery.

## 1. Introduction

Tuberculosis (TB) remains a leading global cause of illness and death. The World Health Organization (WHO) estimated 10.6 million incident TB cases in 2021, and its End TB Strategy targets a 95% reduction in TB deaths and 90% reduction in incidence by 2035. Strengthening diagnosis, prevention, and treatment is essential to achieving these goals.^1^ Despite decades of research and programmatic control efforts, TB remains one of the leading causes of death from infectious diseases. Approximately one-quarter of the world’s population is estimated to be infected, with the BRICS countries—Brazil, Russia, India, China, and South Africa—together accounting for a significant share of new TB cases annually.^2,3^

The WHO’s End TB Strategy set ambitious milestones through 2035; however, progress has been constrained by prolonged treatment courses, adherence challenges, and drug resistance.^4^ TB treatment remains demanding for patients, and real-world experience highlights the social and economic hardships, stigmatization, and household disruptions that accompany the disease across diverse health-system contexts. In BRICS settings specifically, common patient-reported challenges include loss of income, care delays driven by stigma, and variable access pathways across public and private sectors.^2,5^

India accounts for the largest share of the global TB burden in the WHO South-East Asia Region—approximately 28% of global TB—which is among the highest TB-infection burden worldwide. The Indian National TB Prevalence Survey reported a crude TB-infection prevalence of approximately 31% among individuals aged 15 years and above.^1^

Central nervous system (CNS) TB, although less common than pulmonary disease, poses particular diagnostic challenges because its clinical and radiologic manifestations overlap with neoplastic, demyelinating, and vascular conditions. In high-burden settings, these challenges are compounded by the same access, adherence, and stigma-related barriers that affect pulmonary TB care.^2^ Tuberculomas constitute a small fraction of CNS TB cases, with brainstem involvement being particularly rare and accounting for approximately 5% of cases.^6,7^ Clinical manifestations can mimic neoplasms, abscesses, or demyelinating lesions.^7,8^

This case was managed at a tertiary-care government teaching hospital in India, which serves a predominantly mixed rural–urban catchment population where patients often navigate between public services and private practitioners. This pattern is consistent with a prior work describing the diversity of care pathways and health-system challenges in BRICS countries, including India.^3^

## 2. CASE PRESENTATION

A previously healthy female in late adolescence presented to the emergency department (ED) with a one-month history of intermittent holocranial, dull-aching, non-pulsatile headaches. Two weeks after the onset of headache, she developed gradually progressive left-sided limb weakness, initially distal (involving grip strength and foot dorsiflexion) and later progressing proximally over the following two weeks. She also developed difficulty walking unassisted and increasing clumsiness on the left side. By the third week after headache onset, she developed dysarthria, nasal speech, and difficulty swallowing both solids and liquids. Her family also noticed deviation of the mouth to the left and the tongue to the right, which prompted them to seek care in the ED approximately one month after the initial onset of headache.

On examination, she was fully conscious and oriented. Cranial nerve examination revealed: (1) right-sided lower motor neuron (LMN) facial palsy; (2) absent gag reflex with uvula deviation to the left (cranial nerves IX and X involvement); and (3) tongue deviation to the right with atrophy (cranial nerve XII involvement).

Motor examination showed: (1) left-sided spastic hemiparesis (proximal power 3/5, distal 2/5) in both upper and lower limbs; (2) exaggerated deep tendon reflexes; and (3) upgoing plantar response on the left. The right-sided limbs were normal.

No meningeal signs were present. The fundus was normal, and vital signs were as follows: pulse rate 86 beats/min with normal rhythm and volume, and no abnormal pulse character noted. Blood pressure was 104/68 mmHg, measured in the right brachial artery in the supine position. There was no seizure, fever, or altered sensorium. These findings raised suspicion for a lesion affecting the right ventral pons and midbrain, consistent with a “crossed syndrome.”

## 3. INVESTIGATIONS

Routine blood laboratory investigations revealed microcytic, hypochromic anemia and a low total serum protein level (5.2 g/dL). The cerebrospinal fluid (CSF) was clear in appearance, with biochemical analysis showing protein 40 mg/dL, glucose 68 mg/dL, chloride 740 mg/dL, a cell count of 2 cells/cumm, negative Gram and Ziehl–Neelsen stains, adenosine deaminase (ADA) 4.1 U/L, and a negative cartridge-based nucleic acid amplification test (CBNAAT) (Table 1).

**Figure 1.**
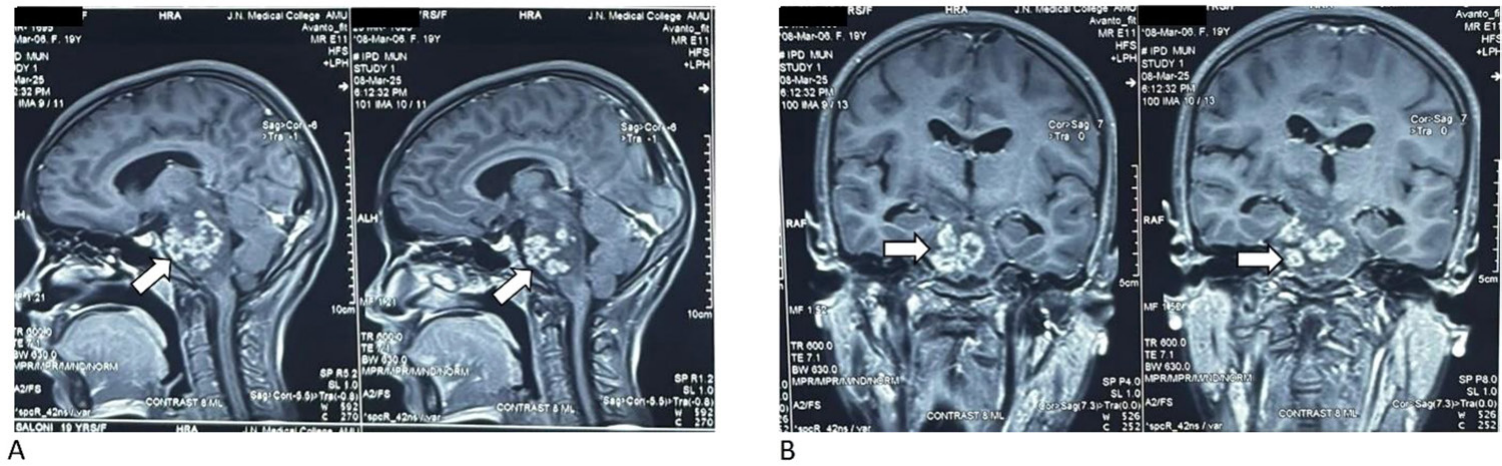
Post-contrast T1-weighted MRI of the brainstem. (A) Sagittal post-contrast T1-weighted images showing conglomerate ring-enhancing lesions (white arrows) in the right midbrain and pons, with central hypointensity and peripheral enhancement. There is an associated mass effect with compression of the aqueduct of Sylvius and effacement of the prepontine cistern. (B) Coronal post-contrast images confirming the location of the lesions within the brainstem (white arrows), demonstrating peripheral enhancement and internal heterogeneity consistent with caseating granulomatous inflammation.

**Figure 2.**
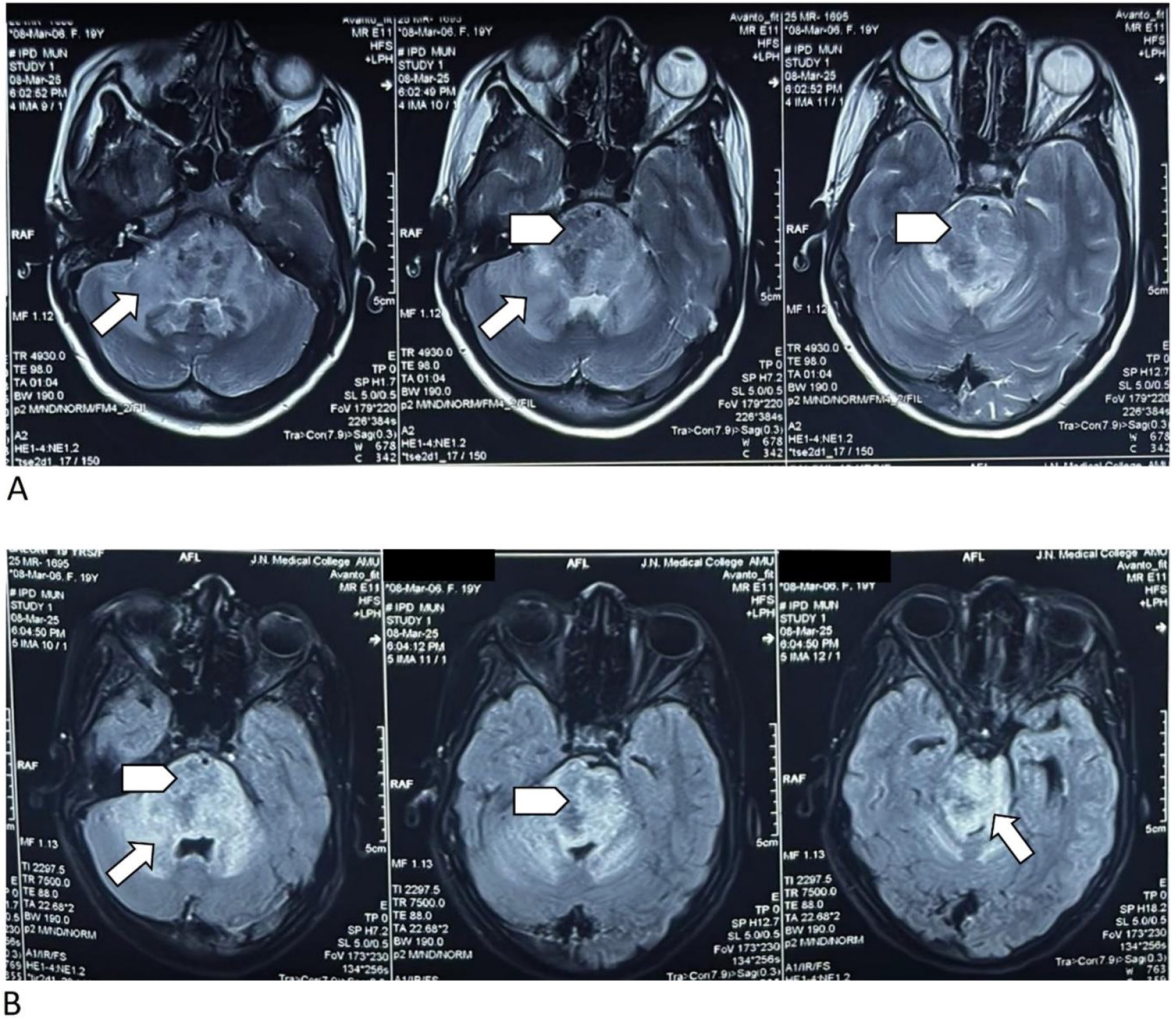
T2-weighted and FLAIR axial MRI brain sequences showing brainstem tuberculoma with surrounding edema (arrows and arrowheads). (A) Axial T2-weighted images showing a well-defined, conglomerated hypointense lesion in the right midbrain and pons (arrowhead), with marked perilesional hyperintensity consistent with vasogenic edema (arrows). The lesion causes mass effect with distortion of the fourth ventricle and mild effacement of perimesencephalic cisterns. (B) Corresponding FLAIR axial images showing the lesion (arrowhead) as heterogeneous hyperintensity, with marked perilesional edema (arrows) extending along the brainstem and posterior fossa.

**Figure 3.**
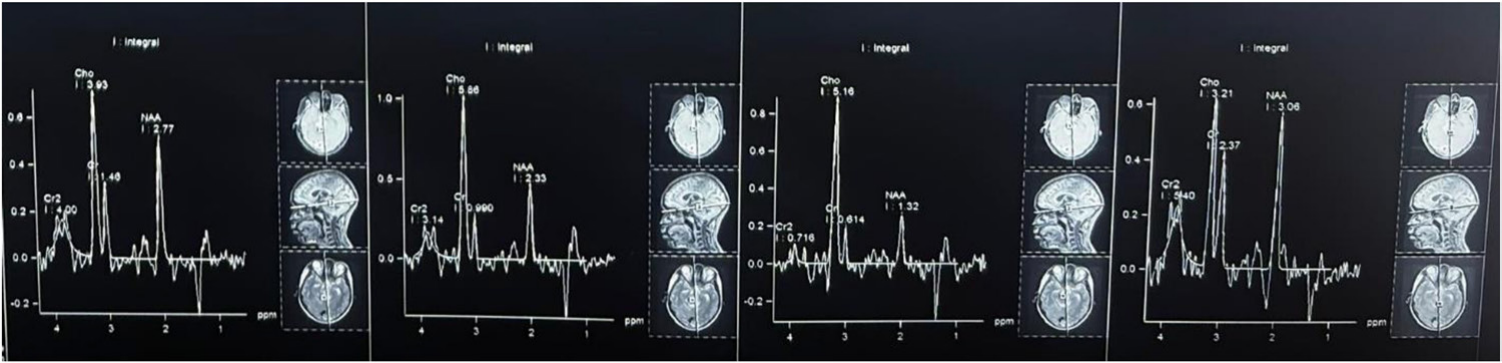
Magnetic resonance spectroscopy (MRS) from different voxels within and around the brainstem lesion. Panels represent MRS spectra acquired from multiple voxels of the lesion and surrounding tissue. Panel 1: Markedly elevated choline (Cho) peak and reduced N-acetylaspartate (NAA) with a lipid–lactate peak at approximately 1.3 ppm, suggestive of necrotic inflammation. Panel 2: Similar spectral profile with elevated Cho and reduced NAA, indicating active granulomatous inflammation. Panel 3: Dominant lipid–lactate peak, severely reduced NAA, and suppressed creatine, typical of the caseating tuberculoma core. Panel 4: Spectra from adjacent parenchyma showing near-normal metabolite ratios, used as an internal control.

Magnetic resonance imaging (MRI) of the brain revealed multiple conglomerated ring-enhancing lesions in the midbrain and pons, with T1/T2 hypointensity and peripheral fluid-attenuated inversion recovery (FLAIR) hyperintensity (Figures 1 and 2). Susceptibility-weighted imaging (SWI) sequences showed blooming foci, while DWI showed no restriction. There was marked adjacent T2/FLAIR hyperintensity consistent with perilesional edema involving the midbrain, pons, bilateral superior and middle cerebellar peduncles, adjacent cerebellar hemispheres, right basal ganglia, and bilateral optic radiation (Figure 2). MRS demonstrated a lipid–lactate peak, with reduced N-acetylaspartate and elevated choline, favoring a necrotic granulomatous process over a tumor (Figure 3).^6,8^ There was associated mass effect causing effacement of the prepontine cistern and narrowing of the cerebral aqueduct, with mild ventricular dilatation (Figures 1 and 2).

## 4. DIFFERENTIAL DIAGNOSIS

Initial differentials included brainstem glioma (ruled out by MRS and the lack of progressive mass), demyelinating disease (no enhancing active plaques or spinal involvement), neurocysticercosis (less likely due to lesion location and MRS findings), infectious granulomas such as tuberculoma, and young stroke (timing and MRS pattern not consistent). The imaging pattern, combined with the endemic context, supported a diagnosis of brainstem tuberculoma (Figure 1).

## 5. TREATMENT

A four-drug anti-tubercular therapy (ATT) regimen was initiated: isoniazid (5 mg/kg), rifampicin (10 mg/kg), pyrazinamide (25 mg/kg), and ethambutol (15 mg/kg). Intravenous dexamethasone was administered at 0.4 mg/kg/day for two weeks, followed by a switch to oral dexamethasone at a dose of 0.2 mg/kg for the third week and 0.1 mg/kg for the fourth week, then gradually tapered over the subsequent four weeks. The patient showed mild improvement within two weeks, with better swallowing and improved left limb strength (now 4/5 proximally). She was discharged on ATT and oral dexamethasone and was scheduled for neurology and imaging follow-up at one month and six months, respectively.

## 6. OUTCOME AND FOLLOW-UP

At the two-month follow-up, she had resumed assisted walking and could tolerate a normal diet. Cranial nerve deficits had partially improved, and power in the left limbs had increased to 4+/5.

## 7. DISCUSSION

Isolated brainstem tuberculoma is an uncommon manifestation of CNS TB and frequently masquerades as a neoplasm, demyelination, abscess, or even brainstem infarction, which can delay diagnosis and treatment.^6–8^ In our patient, the stepwise evolution from headache to a crossed brainstem syndrome (contralateral long-tract signs with ipsilateral cranial neuropathies) localized the lesion to the right pons and medial medulla, prompting targeted imaging.^9^ MRI of the brain demonstrated multiple conglomerated ring-enhancing lesions with extensive perilesional edema, and MRS showed a dominant lipid–lactate peak with reduced N-acetylaspartate—features that favor a caseating granulomatous process over tumor or pyogenic abscess.

Sadashiva et al. highlighted the wide clinical spectrum—from cranial neuropathies and long-tract signs to ataxia—and emphasized that many patients lacked microbiological confirmation, relying instead on a clinico-radiological diagnosis, especially when lesions were deep or posed a high risk for biopsy. Favorable outcomes were common with timely ATT, particularly when the diagnosis was made before irreversible deficits developed. Our patient’s presentation, normal CSF, characteristic MRI/MRS pattern, and good response to medical therapy closely mirror these observations.^6^

Talamás et al. analyzed brainstem tuberculoma cases and similarly noted frequent diagnostic confusion with tumors, emphasizing the importance of integrating endemic context with imaging to justify empirical therapy when biopsy posed undue risk. The clinical trajectory in our patient—progressive deficits followed by improvement after ATT—aligns with the treatment-response pattern summarized in that series.^7^

Yeat et al. described a “tumor-like” brainstem tuberculoma in which the initial radiologic impression favored neoplasm; subsequent clinico-radiological reassessment led to ATT with clinical improvement. This underscores how spectroscopy (lipid–lactate peak) and the absence of diffusion restriction can help avert unnecessary surgery—precisely the scenario in our case.^8^

Parija et al. reported Weber syndrome secondary to brainstem tuberculoma, illustrating that crossed cranial nerve–long–tract patterns are not limited to vascular etiologies. Our patients’ crossed signs (LMN facial palsy, bulbar involvement, contralateral spastic hemiparesis) echo this phenotype and support tuberculoma as an important differential in young patients from endemic regions.^9^

Across studies, three themes recur: (1) CSF is often non-diagnostic in parenchymal brainstem disease; a negative smear/CBNAAT does not exclude tuberculoma, and our patient’s CSF profile fits this pattern. (2) MRI/MRS patterns carry high diagnostic weight—ring enhancement with surrounding edema, lack of diffusion restriction, susceptibility “blooming,” and a lipid–lactate peak on spectroscopy favor tuberculoma over glioma or pyogenic abscess. Our patient’s imaging showed each of these features. (3) Biopsy is reserved for atypical, progressive, or treatment-refractory lesions, given the eloquence and surgical risks of the brainstem—an approach mirrored in our management and prior series.

The literature consistently supports empirical ATT in strongly suspected cases, often with adjunctive corticosteroids to reduce perilesional edema and mass effect, especially when hydrocephalus or cranial nerve dysfunction is present. Similar to previously reported cases, our patient was started on a standard four-drug regimen with a tapering course of steroids and demonstrated early improvement in bulbar function and limb strength within two weeks, with continued gains by two months.^6–9^

For clinicians in TB-endemic settings, these reports—taken together with our case—suggest a pragmatic algorithm: in a young patient with crossed brainstem signs, characteristic MRI/MRS features, and non-diagnostic CSF, it is reasonable to commence ATT with close clinical and imaging follow-up, reserving biopsy for atypical courses or lack of response. This approach may prevent delay-related disability and avoid hazardous brainstem procedures while still allowing timely escalation if the disease behaves atypically.

## 8. CONCLUSION

Brainstem tuberculomas may closely resemble brainstem infarcts and neoplasms at presentation. When considered alongside conventional MRI of the brain, MRS is particularly helpful in distinguishing tuberculoma from glioma or pyogenic abscess. Importantly, a normal CSF profile does not exclude CNS TB. In patients with a compatible clinico-radiologic picture, timely empirical ATT—often with adjunctive corticosteroids—can avert hazardous brainstem biopsy and improve outcomes. In our patient, after a presumptive diagnosis of brainstem tuberculoma was made, ATT with adjunctive corticosteroids was initiated, resulting in early neurological improvement, as evident at the two-month follow-up.

## ETHICAL CONSIDERATION

Written informed consent for the publication of clinical details and images was obtained from the patient and their guardian. The documentation is available with the corresponding author. Ethical approval was obtained from the Institutional Ethics Committee (Ref No. IECJNMC/1610).

## COMPETING INTERESTS

The authors have no conflicts of interest to declare.

## AUTHOR CONTRIBUTIONS

MSA: Conceptualization, methodology, investigation, data curation, writing – original draft, writing – review & editing. KSZ: Methodology, formal analysis, investigation, writing – review & editing, supervision. RK: Investigation, resources, review & editing. All authors have read and approved the final version of the manuscript.

## Figures and Tables

**Table 1.** Cerebrospinal fluid analysis findings on admission in a young female with crossed brainstem syndrome.

Color	Colorless
Appearance	Clear
Protein	40 mg/dL
Sugar	68 mg/dL
Chloride	740 mg/dL
Total cell count	2/cumm
Gram stain	No organisms
Ziehl–Neelsen stain	No organisms
Adenosine deaminase	4.1 U/L
CSF CBNAAT	Negative
